# Supplementary material for: Predicting Silent Atrial Fibrillation in the Elderly: A Report from the NOMED-AF Cross-Sectional Study
Source: J Clin Med. 2021 May 26;10(11):2321. doi: 10.3390/jcm10112321 (PMC8199269; doi:10.3390/jcm10112321)
Supplement: Supplementary file 1 [file jcm-10-02321-s001.zip › jcm-1221493-supplementary.pdf]

Supplementary Table S1. Definitions of medical conditions included to risk factor analysis if they were not established on the basis on survey and medical records review.

Name	Abbreviation	Definition
Arterial Hypertension	HA	Average blood pressure values from two measurements during each visit equal to or higher than 140 mmHg (systolic) and/or 90 mmHg (diastolic), or an individual is taking hypotensive drugs over the past 2 weeks because of an earlier diagnosis of hypertension, as per the 2018 ESC/European Society of Hypertension guidelines [7].
Diabetes	DM	Hemoglobin A1c was ≥6.5%, or if an individual is aware of their diabetes and was taking glucose-lowering agents, in accordance with the 2019 American Diabetes Association and 2019 ESC/European Association for the Study of Diabetes criteria [9,10].
Chronic Kindey Disease	CKD	Estimated glomerular filtration rate <60 mL/min/1.73m2 or ≥60 mL/min/1.73 m2 with coexisting albuminuria (albumin-tocreatinine ratio ≥30 mg/g) using the 2009 Chronic Kidney Disease Epidemiology Collaboration formula [10-12].
Thyroid diseases		Individual aware of any thyroid disease and/or taking of any thyroid hormone supplementation or thyroid suppressing drugs or level of thyroid hormones out of range
